# Emotions and Strategic Behaviour: The Case of the Ultimatum Game

**DOI:** 10.1371/journal.pone.0158733

**Published:** 2016-07-06

**Authors:** Ignacio Tamarit, Angel Sánchez

**Affiliations:** 1 Universidad Carlos III de Madrid, Grupo Interdisciplinar de Sistemas Complejos (GISC), Departamento de Matemáticas, 28911 Leganés, Madrid, Spain; 2 Universidad Carlos III de Madrid, Grupo Interdisciplinar de Sistemas Complejos (GISC), Departamento de Matemáticas, and Institute UC3M-BS of Financial Big Data, 28911 Leganés, Madrid, Spain; 3 Universidad de Zaragoza, Instituto de Biocomputación y Física de Sistemas Complejos (BIFI), 50018 Zaragoza, Spain; University of Reading, UNITED KINGDOM

## Abstract

Human behaviour in economic interactions has attracted an increasing amount of attention over the last decades. The economic assumption that people would behave focusing on their own material self-interest was proved incomplete, once the empirical evidence consistently showed that many other motives may influence such behaviour. Therefore, models that can incorporate rational decision process as well as other intervening factors are a key issue to both understand the observations from economic experiments and to apply the lessons learned from them. In this paper, we incorporate the influence of emotions to the utility function in an explicit manner, using the Ultimatum Game as a case study. Our model is amenable to analytical study, and is connected with the Circumplex model of emotions and with Kahneman’s two-system theory. The simplicity of the model allows to obtain predictions for the offers and acceptance thresholds. We study two specific examples, when the model parameters are distributed uniformly or normally, and show that in the latter case the results are already qualitatively correct. Although this work can be considered as a first approach, it includes what we believe are the main stylized facts, is able to qualitatively reproduce experimental results in a very simple manner, and can be straightforwardly extended to other games.

## Introduction

Experimental economics [1] and, more recently, emerging fields such as neuroeconomics [2] have led to fundamental changes in the understanding of how humans make decisions in economic contexts. Traditionally, economic models assumed that people focus on their own material self-interest when involved in strategic interactions, but the scientifically sound evidence arising from experiments suggests that many other motives may influence behavior [3]. Insights into the nature of the different behaviors obtained from experiments would clearly be relevant in many economic settings, such as consumer response, types of taxation or price formation and change. To that end, formal models that incorporate a variety of motivations for people’s decisions are needed, in so far as they are capable to explain (and possibly predict) how we behave under different circumstances.

Indeed, it is certainly the case that some individuals do behave in a self-regarding manner, and therefore a theory attempting to explain human decision making should leave room for heterogeneity [4]. On the other hand, it has been shown that theoretical approaches can work very well at the aggregate level while performing worse at the individual level [5, 6]. Therefore, models that can incorporate rational decision process as well as other intervening factors are a key issue to both understand the observations from economic experiments and to apply the lessons learned from them. Two main types of models have been proposed so far: one based on bounded rationality ideas, i.e., that the cognitive capabilities of individuals are limited and render them unable to compute their best option (see, e.g., [7, 8]), and an alternative one incorporating social preferences to rational considerations (such as reciprocity [9], inequity aversion [10] or several factors at a time [11]; see also [3] for a comprehensive review).

In this paper, we introduce a new approach to this issue by incorporating emotions to the utility function in an explicit manner, using the Ultimatum Game (UG) as a case study. We choose this specific application because of the wealth of experimental results about it [3] and of the well established fact that many people do not play this game in a material self-interested manner, which makes it a very appropriate testbed for approaches beyond monetary utility. In the UG a fixed amount of money is split between two players: a proposer (*P1*) and a responder (*P2*). *P1* decides what the actual split is and *P2* determines whether it is accepted (and both players share the money as agreed) or rejected (and both players receive nothing). When the game is analyzed from the perspective of classical game theory, three simple assumptions are generally made on the behaviour of the players and their ability to find rational solutions according to their preferences [12]:

- *A1*: Players behave as income-maximizers, and therefore they prefer *α* to *β* whenever *α* > *β* (and they are indifferent over *α* = *β*).
- *A2*: Both players are aware of the condition above.
- *A3*: *P1* can calculate the optimal offer.

Following these assumptions, *P2* should always accept any non-negative payoff rather than nothing (*A1*) and since *P1* knows that (*A2*), he can use backwards induction (*A3*) and offer the smallest possible positive share, which is then accepted by *P2*. That is the so called *Subgame Perfect Nash Equilibrium* of the game [13, 14].

However, as stated above, very many experiments have been performed on UG’s ([3, 4, 15–17]), and the results differ significantly from those predicted by the arguments shown above. Interesting enough is the fact that offers below 20 percent are very rare and they are rejected about half of the times. Modal and median offers are usually 40-50 percent, means are 30-40 percent and there are virtually no offers above the 50 percent split [3, 10]. The robustness of this results has also been tested under cross-cultural perspectives [18]. If we are willing to use game theory as a theoretical framework to explain these results, we must therefore admit that the assumptions from which we derived the previous equilibrium do not correspond with the behaviour of actual players. Clearly, *A1* is proved wrong when confronted with the experimental results, since *P2* does not always prefer a positive payoff rather than a zero payoff. The fact that proposers do offer more than the minimum possible implies that they do not follow *A2*, and hence do not think of others as pure income-maximizers. Given these circumstances it is reasonable to discard as well *A3* on the basis that the idea of an optimal offer is ill-defined. In fact, although *P1* may try to estimate what his best strategy should be, it is impossible for him to find it if both *A1* and *A2* are not true.

In what follows, we show how an approach based on a simple description of emotions offers insights on the observed behavior which, importantly, can not be obtained from alternative descriptions introduced earlier. In order to present our results, the remainder of the manuscript is organized as follows: first, we critically review the available theories proposed to understand behavior that is not well described by the axioms above. Subsequently, we present our model, starting from the implementation of the description of emotions to proceed to the corresponding utility function. We then study our model in detail, obtaining general results and examples arising from specific choices for the model parameters. We conclude with a discussion of our results in view of the available evidence and of the pre-existing theories.

## 1 Earlier work

As we indicated above, the robustness of the experimental results has encouraged the development of several different models. Among these, we will briefly review here those that can be connected to emotions in one way or another, in order to properly frame the contributions of our own approach. Let us begin by discussing the paper by Fehr and Schmidt [10] who, in 1999, proposed a general model (*A Theory of Fairness, Competition, and Cooperation*) in which they included other-regarding preferences in the utility function. For the two-player version of this model, they define *player i’s* utility for the allocation *x* = {*x*_*i*_, *x*_*j*_} as:

$$\begin{array}{c} U_{i}\left(x\right)=x_{i}-\alpha_{i}\max\left(x_{j}-x_{i},0\right)-\beta_{i}\max\left(x_{i}-x_{j},0\right),\;i\neq j \end{array}$$

where they assume that *β*_*i*_ ≤ *α*_*i*_ and 0 ≤ *β*_*i*_ < 1.

The choice *β* ≥ 0 is based on the not-self-evident assumption that nobody likes to be better off than the others, while *β* < 1 implies that a player is not willing to throw away money in order to reduce his advantage relative to other player. Parameters *α* and *β* can be understood as *envy* and *guilt* weights respectively. Indeed, the former reduces utility when the other player’s payoff is greater than one’s payoff, while the latter reduces utility if the focal player’s payoff is greater than the other’s. In the characterization of the parameters, the assumption *α* ≥ *β* implies that players *suffer* more from inequality that is to her disadvantage, and less if it is to her advantage. In our understanding of this approach, it appears that the introduction of these parameters is motivated by how players react emotionally to different allocations. Under this perspective the model represents players’ choices as a combination of income maximization moderated by an emotional rejection (aversion) to inequality. However, there is no explanation for the mechanism behind this reaction, and each individual is characterized by her *envy* and *guilt* parameters without further connection to her emotional mechanisms at work. Notice also that in this model players are not able to know accurately what the preferences of others are, in so far as they don’t know the value of their parameters. For that reason, proposers have to overcome several problems in order to estimate an optimal offer. For instance, she must assume that the other player is also influenced by *envy* and *guilt* (although in an unknown way) and try to estimate her *minimum acceptable offer* (MAO) with this incomplete information.

A different approach was proposed by Rabin [9], who developed a theory of *fairness equilibria* for two-player games in normal form. His model is motivated by the fact that people behave *nicely* to those who treat them nicely and punish those who are not nice to them, with both motivations having a greater effect on behavior as the material cost of sacrificing becomes smaller. Such model includes a representation of subjective judgements and *beliefs* of the players, based on the psychological games framework of Geanakoplos [19]. In order to analyze this model [3] [20], let *a*_*i*_ be the strategy chosen by player *i*, *b*_*j*_ player *i*’s belief about the strategy chosen by player *j*, and *c*_*i*_ player *i*’s belief about the player *j*’s belief about the strategy chosen by *player i*. Then, the utility function (social preference) is defined as:

$$\begin{array}{c} U_{i}\left(a_{i},b_{j},c_{i}\right)=\pi_{i}\left(a_{i},b_{j}\right)+{\tilde{f}}_{j}\left(b_{j},c_{i}\right)+{\tilde{f}}_{j}\left(b_{j},c_{i}\right)f_{i}\left(a_{i},b_{j}\right) \end{array}$$

where *π*_*i*_(*a*_*i*_, *b*_*j*_) is the monetary payoff to player *i*, $f_{i}\left(a_{i},b_{j}\right)=\left(\pi_{j}\left(b_{j},a_{i}\right)-\pi_{j}^{fair}\left(b_{j}\right)\right)/\left(\pi_{j}^{max}\left(b_{j}\right)-\pi_{j}^{min}\left(b_{j}\right)\right)$ is player *i*’s kindness toward player *j*, ${\tilde{f}}_{j}\left(b_{j},c_{i}\right)=\left(\pi_{i}\left(c_{i},b_{j}\right)-\pi_{i}^{fair}\left(c_{i}\right)\right)/\left(\pi_{i}^{max}\left(c_{i}\right)-\pi_{1}^{min}\left(c_{1}\right)\right)$ is the perceived kindness by player *i* with respect to how she is being treated by player *j*, $\pi_{j}^{max}\left(b_{j}\right)$ and $\pi_{j}^{min}\left(b_{j}\right)$ are respectively the highest and lowest possible payoffs for player *j*, and $\pi_{j}^{fair}\left(b_{j}\right)$ is an equitable fair payoff defined as the average of the highest and lowest payoffs.

In order to make the model more tractable and define a *fairness equilibrium*, Rabin assumes that players are willing to maximize their social utilities and that all higher-order beliefs match actual behavior (i.e *a*_*i*_ = *b*_*j*_ = *c*_*i*_) [3]. Again, from our viewpoint such an equilibrium concept is built upon the emotional response of the players (how kindly they feel they are treated) and the assumption that players know with certainty the beliefs of other players, what seems very unrealistic in our opinion. Much like in the discussion above, this model lacks a description and characterization of the mechanisms behind the emotional responses. It is also important to note the intuitive idea of a *fair* payoff being defined as the average of the maximum and minimum, as this point will become relevant later in our discussion.

Finally, another, more recent approach that explicitly points to emotions in the development of an utility function is that of Cox et al [20]. They include a parameter *θ*(*r*, *s*) to represent the emotional state of a given player, as the willingness to pay own for other’s payoff at an allocation on the equal line *x*_*i*_ = *x*_*j*_. It is introduced as an increasing function on both reciprocity (*r*) and the status (*s*), and they assume it to be identical across individuals except for a mean zero idiosyncratic term. The utility for the allocation *x* = {*x*_*i*_, *x*_*j*_} is then defined as:

$$\begin{array}{c} u_{i}\left(x\right)\{\begin{array}{cc} =(x_{i}^{\alpha }+\theta x_{j}^{\alpha })/\alpha & \text{if}\;\;\alpha \in (-\infty ,0)\cup (0,1]\\ =x_{i}x_{j}^{\theta } & \text{if}\;\;\alpha =0 \end{array} \end{array}$$

where *α* is a parameter to be determined experimentally. Once the authors treat the emotion as a function *θ*(*r*, *s*) they are forced to make further assumptions on how reciprocity and status are introduced. It seems unnecessary then to call such a variable *emotional state*, since it remains unclear what the role and mechanism of actual emotions are. Indeed the influence arises from both *r* and *s*, and any inclusion of the term *emotion* appears somewhat artificial and is not necessarily related to any particular theory of emotions. Furthermore, the assumption that *θ*(*r*, *s*) is identical across individuals seems to be very strong and not very realistic.

## 2 Model

### 2.1 Motivation and framework

As we have seen, emotions play a role in the arguments behind different models and explanations of the experimental results. However, none of the models presented so far makes explicit such role in a clear manner. Indeed, the idea that the decision-making process is influenced by emotions is very intuitive. There is also experimental evidence of emotional reactions to unfair offers as measured by skin conductance [21], and of how unfair offers elicit activity in brain areas related to both emotion (anterior insula) and cognition (dorsolateral prefrontal cortex) [22]. In these and many other studies, it is also clear that if the emotion is perceived as negative(anger, frustration, sadness, etc.); as a consequence, it is more likely for a given offer to be rejected [23] [24]. Thus, it seems reasonable to try to understand behavior in UG as a combination of both emotion and cognition, allowing one to explain the experimental results from that departure point.

One of the main contributors to the idea of two different but interacting systems in decision-making processes is nobel laureate Daniel Kahneman [25] [26]. Kahneman posits that such mechanism is governed by the interaction of two different systems, which he calls System 1 and System 2. Processes of System 1 are regarded as fast, effortless, automatic, associative and emotionally charged. System 2, in contrast, is responsible for processes that are slow, controlled, analytical, cognitively demanding and affect free. He suggests four ways in which a judgement or choice may be made [25]:

1. no intuitive response comes to mind, and the judgement is produced by System 2.
2. an intuitive judgement or intention is evoked, and
  - is endorsed by System 2;
  - serves as an anchor for adjustments that respond to other features of the situation;
  - is identified as incompatible with a subjectively valid rule, and blocked from overt expression.

In the context of our attempt to explicitly account for the influence of emotions on behavior, a relevant point is that experimental results show that the average is an easily accesible quantity to System 1 [26]. It is thus tempting to suggest that in an UG, System 1 would rapidly perceive the even split as a benchmark and then trigger an emotional reaction according to the subjective validity (or fairness) of it. System 2 analysis would then correspond to that of a pure income-maximizer player. Interestingly, as discussed above, the average payoff appears as a measure of fairness in Rabin’s approach [9].

**Fig 1:**
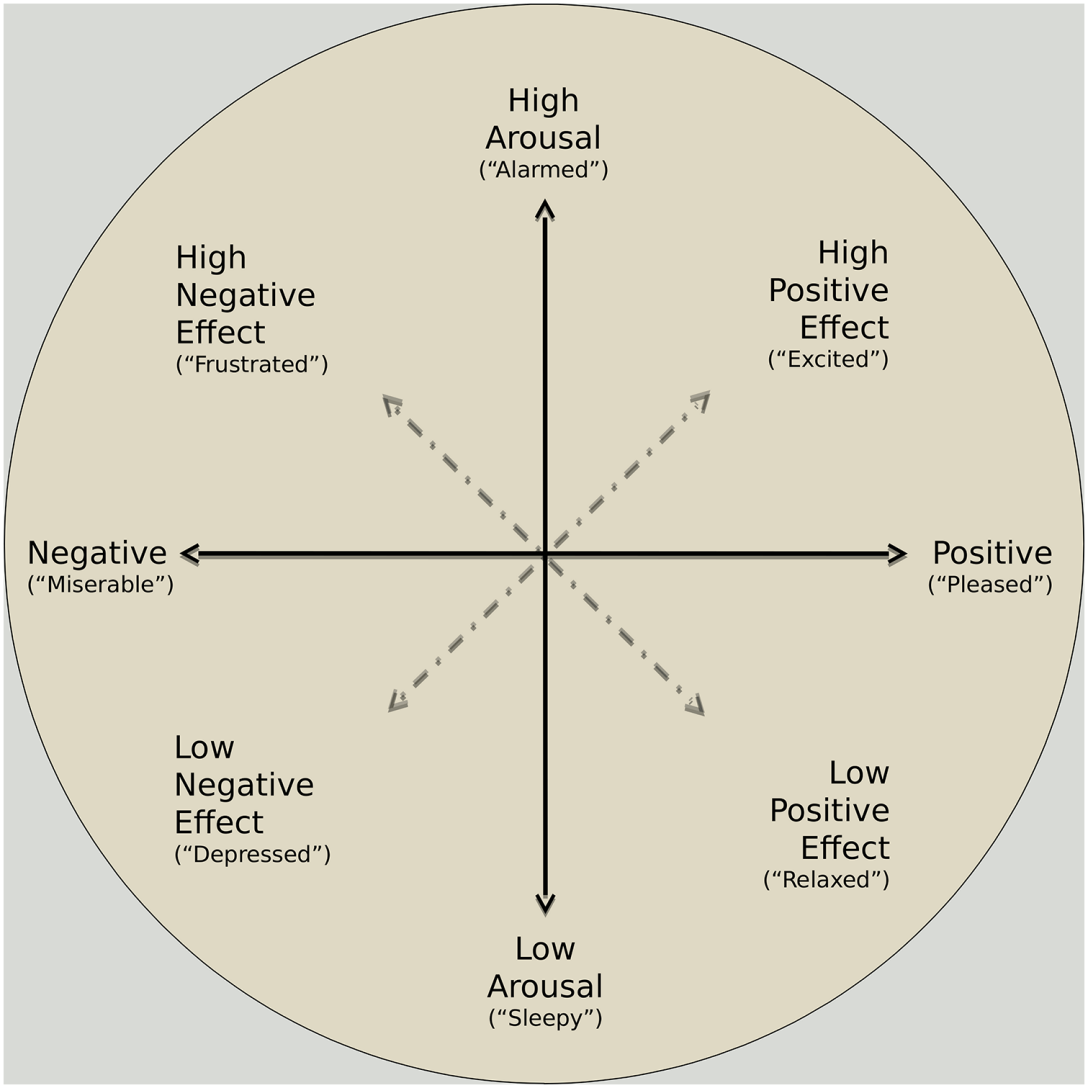
Graphical representation of the circumplex model of emotions. The vertical axis corresponds to the *arousal* dimension and the horizontal one to the *valence*. Each point on the plane represents an emotional state. Sources: [27] [29].

Having found a reasonable starting point for our model, the next step towards defining it is to be able to manipulate the concept of emotion. To that end, we rely on the so called Circumplex Model [27], in which emotions can be categorized in two different continuous dimensions: valence and arousal. Valence indicates whether the pleasure related to an emotion is either positive or negative, while arousal indicates the personal activity induced by that emotion. This emotion model has been used, for instance, by Schweitzer et al. [28] to build an agent-based model of collective emotions in online communities, yielding results resembling actually observed behavior. Fig 1 shows how different emotions may be classified according to this model.

### 2.2 Utility function

In the preceding subsection, we have summarized the two main building blocks of our model. We now move on to its definition by considering the requirements that an utility function should satisfy in order to account for the experimental results, from the viewpoint that the decision making process might be driven by a combination of both emotional and cognitive processes. Therefore, we would like to introduce a model that includes the next facts:

1. Emotions are triggered when offers differ from the perceived average (System 1).
2. The decision making process is a combination of cognitive (System 2) and emotional (System 1) impulses.
3. If a negative emotion (as represented by its *valence*) is triggered then players are willing to give money away in order to compensate for that emotion (as quantified by its *arousal*).
4. Explanatory mechanisms must be compatible with the four ways suggested by Kahneman in which a judgement or choice may be made.

For the sake of simplicity, let us assume that the total amount to be split is equal to one, and let *x*_*i*_ and *x*_*j*_ be the proportions of that amount corresponding to each player (*x*_*i*_ + *x*_*j*_ = 1). Our proposal for player *i*’s utility for an allocation *x* = {*x*_*i*_, *x*_*j*_} is given by

$$\begin{array}{c} u_{i}\left(x\right)=x_{i}+\epsilon \left(x_{i};\lambda_{i},\tau_{i}\right) \end{array}$$ (1)

with

$$\begin{array}{c} \epsilon \left(x_{i};\lambda_{i},\tau_{i}\right)=v\left(x\right)\cdot a\left(x_{i};\lambda_{i},\tau_{i}\right) \end{array}$$ (2)

where

$$\begin{array}{ccc} & & v\left(x\right)=\text{sign}\left(x_{i}-\frac{1}{2}\right)=\{\begin{array}{cc} -1 & \text{if}\;\;x_{i}<1/2\\ 0 & \text{if}\;\;x_{i}=1/2\\ 1 & \text{if}\;\;x_{i}>1/2 \end{array}\\ & & a\left(x_{i};\lambda_{i},\tau_{i}\right)=\lambda_{i}\Theta [|2x_{i}-1\left|-\tau_{i}\right]=\{\begin{array}{cc} 0 & \text{if}\;\;|2x_{i}-1|⩽\tau_{i}\\ \lambda_{i} & \text{if}\;\;|2x_{i}-1|>\tau_{i} \end{array} \end{array}$$

and

$$\begin{array}{c} 0⩽\lambda_{i}⩽1,\;0⩽\tau_{i}⩽1 \end{array}$$

Let us now discuss in detail the ingredients of our model. To begin with, the function *ϵ*(*x*_*i*_; λ, *τ*) represents how an emotion, triggered by the allocation *x*, influences the perceived utility of a player. It can be separated in the product of two quantities; the valence, *v*(*x*), and the arousal, *a*(*x*; λ, *τ*). In agreement with the previously seen Circumplex Model, the former determines whether the emotion is perceived as either positive or negative, and the latter gives account of its intensity in a scale determined by the total amount to be split. Furthermore, the emotion is negative if the amount to consider is less than that of an equal split, and viceversa. The reason behind this choice is that, as we already mentioned, the “average” (the even split in this case) is cognitively easy to evaluate according to Kahneman’s findings [25] [26], and so we take deviations from this pre-stablished value as the baseline to test in which direction may the emotion triggered influence the perceived utility. On the other hand, the arousal *a*(*x*; λ, *τ*) is formulated in terms of a Heaviside function that captures the idea of how this biased thinking may ultimately affect the decision or not. As we have defined it, it implies that deviations from the average must be greater than a parameter *τ* (characteristic of each individual) in order to let the emotion affect the decision making process.

Within the approach we have just introduced, if no emotion is triggered the judgement is entirely determined by System 2. In case that emotions are triggered, this might be enough or not to overcome a purely rational decision. Indeed, if the emotion finally influences the utility function it does so according to parameter λ (characteristic of each individual), which is measured as a percentage of the total economic welfare. Notice that, in contrast with earlier models, this utility function includes emotions as characterized by a psychological model, and its possible outcomes match those suggested by Kahneman.

## 3 Results

### 3.1 General analysis

We are now in a position to start analyzing the game described by our model. To that end, we follow a modified set of assumptions:

- *A1’*: Players behave as utility-maximizers, and therefore they prefer *α* to *β* whenever *u*(*α*) > *u*(*β*) (and they are indifferent over *u*(*α*) = *u*(*β*)).
- *A2’*: Both players are aware of the condition above.
- *A3’*: *P1* uses some *heuristic* to guess *P2’s* preferences and thus calculate her optimal offer.

We note that the first two assumptions are the same as before, only, in the case of *A1’*, referred to our new utility function. The main difference with the previous framework is then that, even if both players want to maximize their respective utilities (and both know that), *P1* has to guess what the MAO of *P2* might be. In order to estimate the MAO of a given player, we believe that a reasonable approach is to find the value for which her utility is minimum but non-negative. From Eq (1) it is immediate to show that *u*_*i*_(*x*_*i*_ < 1/2) < *u*_*i*_(*x*_*i*_ ≥ 1/2), so we must seek for the minimum in offers less than the even split. We are therefore left with:

$$\begin{array}{c} u_{i}\left(x_{i}\right)=\{\begin{array}{cc} x_{i}-\lambda_{i} & \text{if}\;\;x_{i}\in [0,(1-\tau_{i})/2)\\ x_{i} & \text{if}\;\;x_{i}\in [(1-\tau_{i})/2,1/2) \end{array} \end{array}$$ (3)

and, since we are constrained to non-negative utilities, the MAO turns out to be given by:

$$\begin{array}{c} x_{i}^{MAO}=\min\left(\left(1-\tau_{i}\right)/2,\lambda_{i}\right)<1/2 \end{array}$$ (4)

Hence, it is a dominant strategy for the responder to accept any offer $s⩾x_{2}^{MAO}$ and to reject it if $s<x_{2}^{MAO}$. If, on the other hand, the proposer knows the preferences of the responder (given by λ_2_ and *τ*_2_) he will offer (in equilibrium):

$$\begin{array}{c} s^{*}=x_{2}^{MAO} \end{array}$$

In this scenario, nobody has complete information about the possible reactions of other players (*A’3*). Therefore, we proceed to analyze an stylized case. If the proposer does not know the preferences of the responder but believes that *P2’s* preferences are the same as hers, she will offer

$$\begin{array}{c} s_{1}^{*}=x_{1}^{MAO}=\min\left(\left(1-\tau_{1}\right)/2,\lambda_{1}\right). \end{array}$$ (5)

Under the assumptions above, the game is uniquely determined by the distribution of parameters *f*(λ, *τ*). In fact, Eq (5) can be seen as a transformation of the stochastic variables λ and *τ*, and the offer’s distribution, *p*(*s*), can be calculated using that [30]

$$\begin{array}{c} p\left(s\right)=\int_{0}^{1}d\tau \int_{0}^{1}f\left(\lambda ,\tau \right)\delta \left(s-\min\left(\lambda ,\left(1-\tau \right)/2\right)\right)d\lambda \end{array}$$ (6)

where *δ*(*x*) is Dirac’s delta function. Further calculations show that

$$\begin{array}{ccc} p(s) & = & \int_{0}^{1}d\tau \int_{0}^{\frac{1-\tau }{2}}f\left(\lambda ,\tau \right)\delta \left(\lambda -s\right)d\lambda +2\int_{0}^{1}d\tau \int_{\frac{1-\tau }{2}}^{1}f\left(\lambda ,\tau \right)\delta \left(\tau -\left(1-2s\right)\right)d\lambda \\ & = & \int_{0}^{1-2s}f\left(s,\tau \right)d\tau +2\int_{s}^{1}f\left(\lambda ,1-2s\right)d\lambda \end{array}$$ (7)

Therefore, for any given distribution of parameters *f*(λ, *τ*) we can find the corresponding distribution of offers using Eq (7). The same expression also allows us to obtain the probability that an offer 0 < *s* ≤ 0.5 is accepted using the cumulative distribution function: as any player would accept offers greater than her MAO, we have

$$\begin{array}{c} P\left(s\right)=\int_{0}^{s}p\left(r\right)dr \end{array}$$ (8)

The proposer’s expected outcome for a given offer *s*, *g*(*s*), is subsequently given by

$$\begin{array}{c} g(s)=(1-s)P(s) \end{array}$$ (9)

and the offer which maximizes the expected payoff of the proposer, s*, is

$$\begin{array}{c} \frac{dg(s)}{ds}=0\;\Rightarrow \;s^{*} \end{array}$$ (10)

We can take the analysis beyond this point by assuming that the variables λ and *τ* are independent. In this case, we can write *f*(λ, *τ*) = *f*_Λ_(λ)*f*_*T*_(*τ*), and Eq (1) becomes

$$\begin{array}{ccc} p(s) & = & f_{\Lambda }\left(s\right)\int_{0}^{1-2s}f_{T}\left(\tau \right)d\tau +2f_{T}\left(1-2s\right)\int_{s}^{1}f_{\Lambda }\left(\lambda \right)d\lambda \\ & = & f_{\Lambda }\left(s\right)F_{T}\left(1-2s\right)+2f_{T}\left(1-2s\right)\left(1-F_{\Lambda }\left(s\right)\right) \end{array}$$ (11)

where *F*_Λ,*T*_(*x*) are the corresponding cumulative distribution functions. In the particular case that both parameters follow the same distribution *f*(*x*), the former equation can be cast in the form

$$\begin{array}{c} p\left(s\right)=\frac{d}{ds}\left(F\left(1-2s\right)\left(F\left(s\right)-1\right)\right) \end{array}$$ (12)

from which we easily obtain:

$$\begin{array}{c} P(s)=F(1-2s)(F(s)-1)+1 \end{array}$$ (13)

This is the most general result we can obtain (within the assumption of independence of λ and *τ*). In the following subsection, we consider specific examples to assess the ability of our model to explain the observations from experiments.

### 3.2 Examples

In order to illustrate some applications of the previous equations, we use two different distributions for the parameters λ and *τ*: a uniform distribution on the interval λ, *τ* ∈ [0, 1], and a normal distribution *N* [1/2, 1/6]. In the second example the standard deviation is chosen in a way that 99.73% of the values are in the range [0, 1]. With this choice, we neglect any effects produced by values outside the allowed range for our parameters. In both cases, the fact that the distributions are the same for both λ and *τ*, allows us to use Eqs (12) and (13). It has to be stressed that we do not aim at exactly fitting experimental data, but only to show that the model does indeed yield reasonable results as well as to illustrate how, once a distribution for the parameters is obtained, specific predictions arise. Table 1 summarizes the results arising from the two distributions mentioned above in comparison with some robust experimental data [3]:

**Table 1:** Examples and comparison of results with different distributions.

	Uniform	Gaussian	Experimental
Modal offer	∼ 0%	25%	40-50%
Median offer	19%	24%	40-50%
Mean offer	21%	24%	30-40%
Offers in range 1-10%	28%	4%	∼ 0%
Offers in range 50-100%	∼ 0%	∼ 0%	∼ 0%
Rejection of offers in range 40-50%	6%	1%	∼ 0%
Rejection of offers in range 1-20%	48%	70%	50%

As can be immediately seen, the results arising from the uniform distribution exhibit a few important discrepancies with the experimental results, namely the modal offer and the percentage of low offers. On the other hand, the Gaussian distribution gives qualitatively correct results, its main difficulty being the large amount of rejections below 20% of the pot. We note that the rejection of offers in range 40-50% has been estimated as the proportion of individuals that would only accept offers greater than 45%. Notwithstanding the general satisfactory agreement, particularly for the Gaussian distribution, it is evident that in both cases modal, median and mean offers are quantitatively incorrect, lower than those obtained experimentally. This could be at least potentially corrected in an evolutionary framework. In fact, if we apply Eq (10) to find the predicted optimal offer we get 39% for the uniform distribution and 23% for the gaussian. For the uniform distribution, the optimal offer is much higher than the modal, median and mean offers reported in Table 1. In this case, if we consider an evolutionary version of the game, it is clearly possible that the population gets closer to the experimentally observed values, because players using the optimal offer would perform better. On the other hand, the optimal offer for the gaussian distribution is already very close to the central offer values, and therefore even in an evolutionary framework it should not change much.

## 4 Discussion

In this paper, we have introduced a novel manner to account for the influence of emotions on economic decision-making through a modified utility function. In contrast with previous approaches [9, 10, 20], our framework includes explicitly emotional contributions in the utility function expressed in terms of valence and arousal, i.e., following the Circumplex model [27] and making contact with Kahneman’s two-system approach [26]. In our model, valence, the positive or negative charge of the emotion, arises from the way the action of one’s counterpart is perceived, and arousal requires a significant deviation from the expected or desired behavior before emotions take over pure rationality. While we have focused for definiteness on the Ultimatum game, the same ideas can apply to any other game or economic interaction and therefore our proposal is a general one.

In the specific context of the UG, our model is amenable to analytical study and we have thus provided general results for the players’ behavior that depend only on the distribution of our two emotional parameters in the population. In order to illustrate the results arising from our approach, we have chosen two very simple case studies, given by a uniform distribution and a Gaussian one. The uniform distribution does not provide good results, although this is not unexpected because it allows for very different emotional motivations and consequences in the population. The Gaussian distribution already gives qualitatively correct results compared to the experiments, albeit it underestimates the offers and the acceptance levels. It goes without saying that, were more specific information on the possible distributions of the emotional parameters in the population, they could be immediately inserted in our results to obtain specific predictions about the observed behavior. Interestingly, the model predicts that the choice of an amount to split would influence the outcomes if it changes from those typically used (5, 10, etc.) to some other numbers “difficult to average” (i.e 137).

As we have already said, the model presented is a first approach, trying to capture different ideas in decision-making processes and the role emotions play in them. It includes what we believe are the main stylized facts, although it could be enhanced in several different ways to fit experimental data in a more general theory yet to come. It would also be useful to study the application of these ideas to other games to check the validity and accuracy of the corresponding predictions, thus allowing to better understand the influence of emotions in strategic interactions. To be sure, this is a quite subtle and complex problem. Trying to mathematically formalize a theory of emotions seems like a daunting task, but having some insights that help us to understand human behavior can be a more achievable goal. In order to test the validity of the model, the first thing that needs to be done is to determine the distribution of parameters (which should be independent of the game), and test whether it is robust or not. The increasing interest in measuring emotional reactions [31] in different situations is a great opportunity to do so. In fact, if the ideas presented here match experimental data, the extension of the model to other games and situations would be the definitive test.

Finally, a key point in obtaining Eqs (5)–(13) is the assumption that the proposer thinks of others *as if* they were like himself. This heuristic facilitates the analytical calculations, and serves as a first approximation to the problem. As has been pointed out, it is not possible for a player to have complete information on the other players in models that include parametric descriptions of individuals. A more realistic approach would take into account the history of the player in former interactions as a proxy of how others may behave. We hope that this work facilitates further research along these directions.

## References

[1] Smith VL. Microeconomic systems as an experimental science. Am Econ Rev. 1982 12;72(5):923–955.

[2] Glimcher P, Camerer C, Fehr E, Poldrack R, editors. Neuroeconomics: Decision making and the brain. London: Elsevier; 2009 526 pp

[3] Camerer C. Behavioral game theory: Experiments in strategic interaction. Princeton:Princeton University Press; 2003 568 pp

[4] Brañas-Garza P, Espín A, Exadaktylos F, Herrmann B. Fair and unfair punishers coexist in the Ultimatum Game. Sci Rep. 2014 8;4:6025 Available from: 10.1038/srep06025 10.1038/srep06025 25113502 PMC4129421

[5] Kirman AP. Whom or what does the representative individual represent? J Econ Perspec. 1992;6(2):117–136. Available from: 10.1257/jep.6.2.117 10.1257/jep.6.2.117

[6] Blanco M, Engelmann D, Norman HT. A within-subject analyisis of other-regarding preferences. Games Econ Behav. 2011 6;72(2):321–338. Available from: 10.1016/j.geb.2010.09.008 10.1016/j.geb.2010.09.008

[7] Roth AE, Erev I. Learning in extensive form games: Experimental data and simple dynamic models in the intermediate term. Games Econ Behav. 1995;8(1):164–212. Available from: 10.1016/S0899-8256(05)80020-X 10.1016/S0899-8256(05)80020-X

[8] Camerer C, Ho TH. Experienced-weighted attraction learning in normal form games. Econometrica. 1999 7;67(4):827–874. Available from: 10.1111/1468-0262.00054 10.1111/1468-0262.00054

[9] Rabin M. Incorporating fairness into game theory and economics. Am econ rev. 1993 12;83(5): 1281–1302.

[10] Fehr E, Schmidt KM. A theory of fairness, competition, and cooperation. Q J Econ. 1999 8;114(3): 817–868. 10.1162/003355399556151

[11] Charness G, Rabin M. Understanding social preferences with some simple tests. Q J Econ. 2002 8;117(3):817–869. Available from: http://www.jstor.org/stable/4132490 10.1162/003355302760193904

[12] Bearden JN. Ultimatum bargaining experiments: The state of the art. SSRN 2001 Nov. Available from: http://ssrn.com/abstract=626183

[13] Vega-Redondo F. Economics and the theory of games. Cambridge: Cambridge University Press; 2003 528 pp

[14] Gintis H. Game theory evolving. 2nd ed Princeton: Princeton University Press; 2009 408 pp

[15] Güth W, Schmittberger R, Schwarze B. An experimental analysis of ultimatum bargaining. J Econ Behav Organ. 1982 12;3(4):367–388. 10.1016/0167-2681(82)90011-7

[16] Thaler RH. Anomalies: The ultimatum game. J Econ Perspec. 1988;2(4): 195–206. 10.1257/jep.2.4.195

[17] Güth W, Tietz R. Ultimatum bargaining behavior: A survey and comparison of experimental results. J Econ Psychol. 1990 9;11(3):417–449. 10.1016/0167-4870(90)90021-Z

[18] Henrich J, Boyd R, Bowles S, Camerer C, Fehr E, Gintis H, et al Economic man in cross-cultural perspective: Behavioral experiments in 15 small-scale societies. Behav Brain Sci. 2005 12;28(06):795–815. 10.1017/S0140525X05000142 16372952

[19] Geanakoplos J, Pearce D, Stacchetti E. Psychological games and sequential rationality. Game Econ Behav. 1989 3;1(1):60–79. 10.1016/0899-8256(89)90005-5

[20] Cox JC, Friedman D, Gjerstad S. A tractable model of reciprocity and fairness. Game Econ Behav. 2007 4;59(1):17–45. 10.1016/j.geb.2006.05.001

[21] Van’t Wout M, Kahn RS, Sanfey AG, Aleman A. Affective state and decision-making in the ultimatum game. Exp Brain Res. 2006 3;169(4):564–568. 10.1007/s00221-006-0346-5 16489438

[22] Sanfey AG, Rilling JK, Aronson JA, Nystrom LE, Cohen JD. The neural basis of economic decision-making in the ultimatum game. Science. 2003 6;300(5626):1755–1758. 10.1126/science.1082976 12805551

[23] Bosman R, Sonnemans J, Zeelenberg M. Emotions, rejections, and cooling off in the ultimatum game. 2001;Unpublished manuscript, University of Amsterdam.

[24] Dunn BD, Evans D, Makarova D, White J, Clark L. Gut feelings and the reaction to perceived inequity: The interplay between bodily responses, regulation, and perception shapes the rejection of unfair offers on the ultimatum game. Cogn Affect Behav Neurosci. 2012 9;12(3):419–429. 10.3758/s13415-012-0092-z 22618636 PMC3400033

[25] Kahneman D. Maps of bounded rationality: A perspective on intuitive judgement and choice. Nobel prize lecture. 2002 12:351–401. Available at: http://www.nobelprize.org/nobel_prizes/economic-sciences/laureates/2002/kahnemann-lecture.pdf

[26] Kahneman D. A perspective on judgement and choice: Mapping bounded rationality. Am Psychol. 2003 9;58(9):697–720 10.1037/0003-066X.58.9.697 14584987

[27] Russell JA. A circumplex model of affect. J Pers Soc Psychol. 1980;39(6):1161–1178 10.1037/h0077714

[28] Schweitzer F, Garcia D. An agent-based model of collective emotions in online communities. Eur Phys J B. 2010 10;77(4):533–545. 10.1140/epjb/e2010-00292-1

[29] Jonker CS, Van der Merwe A. Emotion episodes of Afrikaans-speaking employees in the workplace: Original research. SAJIP. 2013;39(1):1–12.

[30] Van Kampen NG. Stochastic processes in physics and chemistry, Vol 1 Amsterdam:Elsevier; 1992 480 pp

[31] Phelps EA. The study of emotion in neuroeconomics In: Glimcher PW, Camerer CF, Fehr E, Poldrack RA, editors. Neuroeconomics. Amsterdam:Elsevier; 2009 233–250.

